# Supplementary material for: Value generalization in human avoidance learning
Source: eLife. 2018 May 8;7:e34779. doi: 10.7554/eLife.34779 (PMC5957527; doi:10.7554/eLife.34779)
Supplement: Supplementary file 1. — Unless otherwise specified, figures represent mean (SD). STAI, Spielberger State-Trait Anxiety Inventory (trait score only); AMI, Apathy Motivation Index; OCI-R, Obsessive-Compulsive Index (Revised); PHQ9, Physician’s Health Questionnaire 9 (a brief measure of mood disorder symptoms); BIS-11, Barratt Impulsivity Scale (version 11); CSQ global, Cognitive Style Questionnaire (short-form) ‘cognitive globalisation’ subscale. [file elife-34779-supp1.docx]

|  | **fMRI sample**  **(*N*=26)** |  | **AMT sample**  **(*N*=482)** | **Possible range** |
| --- | --- | --- | --- | --- |
| Age | 25.3 (5.6) |  | 37.2 (11.4) | - |
| Gender (%F) | 13 (50%) |  | 249 (52%) | - |
| STAI total | 42.0 (8.1) |  | 39.9 (12.8) | 20-80 |
| AMI total | - |  | 40.1 (8.5) | 0-72 |
| OCI-R total | - |  | 11.3 (10.8) | 0-72 |
| PHQ9 total | - |  | 4.3 (5.1) | 0-24 |
| BIS-11 total | - |  | 56.6 (10.8) | 30-120 |
| CSQ global | - |  | 29.1 (7.9) | 0-48 |
