# Supplementary material for: Value generalization in human avoidance learning
Source: eLife. 2018 May 8;7:e34779. doi: 10.7554/eLife.34779 (PMC5957527; doi:10.7554/eLife.34779)
Supplement: Supplementary file 2. — STAI, Spielberger State-Trait Anxiety Inventory (trait score only); AMI, Apathy Motivation Index; OCI-R, Obsessive-Compulsive Index (Revised); PHQ9, Physician’s Health Questionnaire 9 (a brief measure of mood disorder symptoms); BIS-11, Barratt Impulsivity Scale (version 11); CSQ, Cognitive Style Questionnaire (short-form). [file elife-34779-supp2.docx]

| **Measure** | **Cronbach’s α** | **Rating** |
| --- | --- | --- |
| STAI | 0.960 | Excellent |
| AMI | 0.720 | Acceptable |
| OCI-R | 0.922 | Excellent |
| PHQ9 | 0.915 | Excellent |
| BIS-11 | 0.876 | Good |
| CSQ (all) | 0.900 | Excellent |
| CSQ internal | 0.726 | Acceptable |
| CSQ global | 0.827 | Good |
| CSQ stable | 0.454 | Unacceptable |
| CSQ self-worth | 0.909 | Excellent |
