# Supplementary material for: Value generalization in human avoidance learning
Source: eLife. 2018 May 8;7:e34779. doi: 10.7554/eLife.34779 (PMC5957527; doi:10.7554/eLife.34779)
Supplement: Supplementary file 3. — Item loadings are only shown above a threshold of ±0.25). Text in square brackets is to aid interpretation of reverse-scored items. [file elife-34779-supp3.docx]

| **Items loading onto Factor 1 (“Intrusive anxiety”)** | **Loading** |
| --- | --- |
| I am upset by unpleasant thoughts that come into my mind against my will | 0.762 |
| I find it difficult to control my thoughts | 0.749 |
| I frequently get nasty thoughts and have difficulty in getting rid of them | 0.725 |
| I get upset if others change the way I have arranged things | 0.682 |
| I check things more often than necessary | 0.673 |
| I get upset if things are not arranged properly | 0.632 |
| I have disturbing thoughts | 0.627 |
| I need things to be arranged in a particular order | 0.624 |
| I get in a state of tension or turmoil as I think over my recent concerns and interests | 0.619 |
| I have racing thoughts | 0.605 |
| Little interest or pleasure in doing things | 0.598 |
| I repeatedly check doors, windows, drawers etc. | 0.593 |
| I sometimes shave or wash or clean myself simply because I feel contaminated | 0.591 |
| Some unimportant thought runs through my mind and bothers me | 0.588 |
| I worry too much over something that doesn’t really matter | 0.575 |
| I feel that difficulties are piling up so that I cannot overcome them | 0.574 |
| Feeling down, depressed, or hopeless | 0.569 |
| I feel I have to repeat certain numbers | 0.564 |
| I have saved up so many things that they get in the way | 0.562 |
| I avoid throwing things away because I am afraid I might need them later | 0.562 |
| I wash my hands more often and longer than necessary | 0.558 |
| I often have extraneous or intrusive thoughts when trying to think | 0.557 |
| Feeling tired or have little energy | 0.555 |
| I wish I could be as happy as others seem to be | 0.550 |
| Trouble concentrating on things such as reading the newspaper or watching television | 0.550 |
| I collect things I don’t need | 0.545 |
| Poor appetite or over-eating | 0.541 |
| I feel nervous and restless | 0.540 |
| I feel compelled to count while I am doing things | 0.540 |
| Feeling bad about yourself or that you are a failure or have let yourself down | 0.539 |
| I take disappointments so keenly I can’t put them out of my mind | 0.534 |
| I find it difficult to touch an object I know has been touched by a stranger | 0.507 |
| I feel like a failure | 0.491 |
| I feel like there are good and bad numbers | 0.490 |
| I repeatedly check gas/water taps and light switches after turning them off | 0.470 |
| I feel inadequate | 0.466 |
| Trouble falling or staying asleep, or sleeping too much | 0.465 |
| Moving or speaking so slowly that other people could have noticed (or the opposite) | 0.429 |
| I am [un]happy | 0.381 |
| I am [not] content | 0.381 |
| I lack self-confidence | 0.370 |
| I [do not] feel secure | 0.362 |
| I feel [un]pleasant | 0.345 |
| I feel [un]satisfied with myself | 0.337 |
| I feel [un]rested | 0.332 |
| I am [not] ‘cool, calm, and collected’ | 0.316 |
| I squirm during plays or lectures | 0.307 |
| I am a[n un]steady person | 0.296 |
| I am restless at the theatre or in lectures | 0.286 |
| I [don’t] like to think about complex problems | -0.264 |
| Getting on badly with my parents is [not] the fault of other people or circumstances | -0.275 |
| I am happy-go-lucky | -0.294 |

| **Items loading onto Factor 2 (“Low self-worth”)** | **Loading** | |
| --- | --- | --- |
| That people were not interested in me at the party [says something] about me as a person | 0.742 |  |
| Getting a negative reaction to my talk [says something] about me as a person | 0.732 |  |
| People not being interested in me at the party means there is something wrong with me as a person | 0.700 |  |
| The reason that the talk went badly [will] stop me from enjoying other things | 0.694 |  |
| I will [not] start afresh in the future and forget about the reason people were not interested in me | 0.659 |  |
| That I’m not in a romantic relationship now [says something] about me as a person | 0.651 |  |
| The reason that this evaluation went badly [will] stop be from enjoying other things | 0.642 |  |
| Getting a negative evaluation on my job performance [says something] about me as a person | 0.632 |  |
| I will [not] start afresh in the future and forget about the reason for the talk going badly | 0.625 |  |
| Doing badly on this talk means that there is something wrong with me as a person | 0.593 |  |
| That I’m not able to get all my work done at the moment [says something] about me as a person | 0.582 |  |
| I will [not] start afresh in the future and forget about the reasons for my evaluation being negative | 0.572 |  |
| I will [not] start afresh in the future and forget about the reason I think I’m not in a relationship at the moment | 0.570 |  |
| The reason why I think other people did not seem interested in me [will] stop me from enjoying other things | 0.558 |  |
| Doing badly on this evaluation means that there is something wrong with me as a person | 0.544 |  |
| The reason why I think I can’t get my work done [will] stop me from enjoying other things | 0.539 |  |
| Getting along badly with my parents [says something] about me as a person | 0.532 |  |
| That I am not in a romantic relationship right now means there is something wrong with me as a person | 0.531 |  |
| It is my fault that people are not interested in me | 0.526 |  |
| The reason I am getting on badly with my parents [will] stop me from enjoying other things | 0.503 |  |
| I will [not] try to start afresh in the future and forget about the reason I think I can’t complete my work at the moment | 0.488 |  |
| That I am not able to complete the work means that there is something wrong with me as a person | 0.486 |  |
| The reason no one was interested in me causes me problems in all areas of my life | 0.475 |  |
| The reason I did badly on my talk causes problems for me in all areas of my life | 0.458 |  |
| I lack self-confidence | 0.447 |  |
| The reason why I think I’m not in a relationship [will] stop me from enjoying other things | 0.413 |  |
| Getting along badly with my parents means there is something wrong with me as a person | 0.407 |  |
| It is my fault the class reacted negatively to my talk | 0.399 |  |
| People not being interested in me is [not] the fault of other people | 0.386 |  |
| I feel inadequate | 0.381 |  |
| The reason causing me not to be in a relationship causes me problems in all areas of my life | 0.380 |  |
| The reason I did badly on my evaluation causes me problems in all areas of my life | 0.367 |  |
| I feel [un]satisfied with myself | 0.347 |  |
| My parents and I will [not] start afresh in the future and forget about the reason for getting along badly | 0.345 |  |
| I feel [un]pleasant | 0.331 |  |
| I am [un]happy | 0.331 |  |
| I feel like a failure | 0.310 |  |
| I wish I could be as happy as others seem to be | 0.287 |  |
| I [do not] feel secure | 0.286 |  |
| I [don’t] enjoy choosing what to do from a range of activities | 0.285 |  |
| I take disappointments so keenly I can’t put them out of my mind | 0.280 |  |
| I am [not] content | 0.275 |  |
| I am [not] ‘cool, calm and collected’ | 0.270 |  |
| The same reason causing me to not complete my work causes me problems in all areas of my life | 0.262 |  |
| I [don’t] like to think about complex problems | 0.259 |  |
| It is my fault that I am not in a romantic relationship | 0.258 |  |
| The negative reaction to my talk is [not] the fault of other people or circumstances | 0.252 |  |
| I act on the spur of the moment | -0.260 |  |
| I am happy-go-lucky | -0.325 |  |
| The reason that I’m not in a relationship now will not prevent me from being in a relationship in the future | -0.459 |  |
| The reason that I can’t get my work done now will not prevent me from being able to get everything done in the future | -0.506 |  |
| The reason that people didn’t seem interested in me at this party will not prevent me from enjoying similar situations in the future | -0.715 |  |

| **Items loading onto Factor 3 (“Low self-control”)** | **Loading** | |
| --- | --- | --- |
| I [don’t] plan tasks carefully | | 0.775 |
| I am [not] a careful thinker | | 0.681 |
| I am [not] self-controlled | | 0.659 |
| I am [not] a careful thinker | | 0.633 |
| I [don’t] plan trips well ahead of time | | 0.627 |
| I [do not] plan for job security | | 0.615 |
| I [do not] concentrate easily | | 0.611 |
| I do things without thinking | | 0.552 |
| I [do not] save regularly | | 0.546 |
| I don’t pay attention | | 0.512 |
| I act on impulse | | 0.506 |
| When I decide to do something I am [not] motivated to see it through to the end | | 0.503 |
| I am an [un]steady person | | 0.484 |
| I am [not] future oriented | | 0.479 |
| I spend more than I earn | | 0.447 |
| I act on the spur of the moment | | 0.438 |
| I [don’t] get things done when they need to be but require [reminders] from others | | 0.433 |
| I make decisions [with difficulty] | | 0.400 |
| I buy things on impulse | | 0.397 |
| I say things without thinking | | 0.363 |
| I am [not] ‘cool, calm and collected’ | | 0.360 |
| I get easily bored when solving thought problems | | 0.350 |
| I am [not] content | | 0.339 |
| When I have something I need to do I [don’t] do it straight away so it is out of the way | | 0.334 |
| When I decide to do something I am [not] able to make an effort easily | | 0.327 |
| I feel [un]satisfied with myself | | 0.323 |
| I often have extraneous or intrusive thoughts when trying to think | | 0.318 |
| I feel [un]pleasant | | 0.312 |
| I [do not] feel secure | | 0.305 |
| I feel [un]rested | | 0.298 |
| If I realise I have been unpleasant to someone I will [not] feel terribly guilty afterwards | | 0.276 |
| I am [un]happy | | 0.273 |
| I change jobs | | 0.269 |
| Trouble concentrating on things such as reading the newspaper or watching television | | 0.267 |
| I [do not] like puzzles | | 0.265 |
| I [don’t] enjoy choosing what do to from a range of activities | | 0.264 |
| I [don’t] like to think about complex problems | | 0.261 |
| I get upset if others change the way I have arranged things | | -0.292 |
| I need things to be arranged in a particular order | | -0.297 |
| I get upset if things are not arranged properly | | -0.310 |
