# Supplementary material for: Value generalization in human avoidance learning
Source: eLife. 2018 May 8;7:e34779. doi: 10.7554/eLife.34779 (PMC5957527; doi:10.7554/eLife.34779)
Supplement: Transparent reporting form [file elife-34779-transrepform.docx]

***eLife’s* transparent reporting form**

We encourage authors to provide detailed information *within their submission* to facilitate the interpretation and replication of experiments. Authors can upload supporting documentation to indicate the use of appropriate reporting guidelines for health-related research (see [EQUATOR Network](http://www.equator-network.org/%20)), life science research (see the [BioSharing Information Resource](https://biosharing.org/)), or the [ARRIVE guidelines](http://www.plosbiology.org/article/info:doi/10.1371/journal.pbio.1000412) for reporting work involving animal research. Where applicable, authors should refer to any relevant reporting standards documents in this form.

If you have any questions, please consult our Journal Policies and/or contact us: [editorial@elifesciences.org](mailto:editorial@elifesciences.org).

**Sample-size estimation**

- You should state whether an appropriate sample size was computed when the study was being designed
- You should state the statistical method of sample size computation and any required assumptions
- If no explicit power analysis was used, you should describe how you decided what sample (replicate) size (number) to use

Please outline where this information can be found within the submission (e.g., sections or figure legends), or explain why this information doesn’t apply to your submission:

Sample sizes were based on power analyses for both groups of participants: please see sections Methods > Design > fMRI sample > Protocol for relevant details for the neuroimaging (fMRI) sample, and Methods > Design > Online sample > Protocol for the online sample.

**Replicates**

- You should report how often each experiment was performed
- You should include a definition of biological versus technical replication
- The data obtained should be provided and sufficient information should be provided to indicate the number of independent biological and/or technical replicates
- If you encountered any outliers, you should describe how these were handled
- Criteria for exclusion/inclusion of data should be clearly stated
- High-throughput sequence data should be uploaded before submission, with a private link for reviewers provided (these are available from both GEO and ArrayExpress)

Please outline where this information can be found within the submission (e.g., sections or figure legends), or explain why this information doesn’t apply to your submission:

For the fMRI sample (*N*=26) no participants were excluded from further analysis

(see Methods > Design > fMRI sample > Protocol; Figure 1 legend).

For the online sample, planned quality control was carried out based on previously published work using the Amazon Mechanical Turk (AMT) interface, resulting in the exclusion of 68/550 datasets (final *N*=482, for full details see Methods > Protocol > Design > Online sample> Quality control procedure; see also Figure 1 legend).

**Statistical reporting**

- Statistical analysis methods should be described and justified
- Raw data should be presented in figures whenever informative to do so (typically when N per group is less than 10)
- For each experiment, you should identify the statistical tests used, exact values of N, definitions of center, methods of multiple test correction, and dispersion and precision measures (e.g., mean, median, SD, SEM, confidence intervals; and, for the major substantive results, a measure of effect size (e.g., Pearson's r, Cohen's d)
- Report exact p-values wherever possible alongside the summary statistics and 95% confidence intervals. These should be reported for all key questions and not only when the p-value is less than 0.05.

Please outline where this information can be found within the submission (e.g., sections or figure legends), or explain why this information doesn’t apply to your submission:

Please see Results for full details of all statistical tests carried out.

For functional imaging data, the statistical approach used (and justification) can be found in the section Methods > Analysis > Functional imaging data > Univariate analysis and Methods > Analysis > Functional imaging data > Multivariate analysis.

For behavioural data from both groups (fMRI and online samples), details of computational model fitting and the model comparison procedure can be found in Methods > Analysis > Instrumental avoidance behavior.

The statistical approach taken in relating individual differences in estimated model parameters and other key variables (multivariate representational similarity in the fMRI sample, self-reported psychopathology in online sample) is explained and justified in the section Methods > Analysis > Individual differences.

(For large datasets, or papers with a very large number of statistical tests, you may upload a single table file with tests, Ns, etc., with reference to sections in the manuscript.)

**Group allocation**

- Indicate how samples were allocated into experimental groups (in the case of clinical studies, please specify allocation to treatment method); if randomization was used, please also state if restricted randomization was applied
- Indicate if masking was used during group allocation, data collection and/or data analysis

Please outline where this information can be found within the submission (e.g., sections or figure legends), or explain why this information doesn’t apply to your submission:

N/A (no group allocation was carried out in this study)

**Additional data files (“source data”)**

- We encourage you to upload relevant additional data files, such as numerical data that are represented as a graph in a figure, or as a summary table
- Where provided, these should be in the most useful format, and they can be uploaded as “Source data” files linked to a main figure or table
- Include model definition files including the full list of parameters used
- Include code used for data analysis (e.g., R, MatLab)
- Avoid stating that data files are “available upon request”

Please indicate the figures or tables for which source data files have been provided:

All relevant code for stimulus generation, data collection, and data analysis, in addition to raw behavioural data, is available at the project’s Open Science Framework page (osf.io/25t3f)*.* Raw functional imaging data is deposited at openfMRI (openfmri.org/dataset/ds000249) and derived statistical maps are available at NeuroVault (neurovault.org/collections/3177).

(Methods > Code and data availability)
